# Prosthetic Mitral Valve Endocarditis Complicated by Left Ventricular Pseudoaneurysm

**DOI:** 10.7759/cureus.40513

**Published:** 2023-06-16

**Authors:** Maxwell L Todd, Atul Eppurath, Ramy Shoela

**Affiliations:** 1 Radiology, Saint Louis University School of Medicine, St. Louis, USA

**Keywords:** pseudoaneurysm, ventricular aneurysm, false aneurysm, infective endocarditis, cardiac defect, cardiac implantations, ct cardiac, focused cardiac ultrasound, body and cardiac ct, pet scans

## Abstract

Left ventricular pseudoaneurysm is a rare complication that can result from mitral valve replacement. Proper follow-up imaging can help to detect this potentially fatal complication and identify areas of concern. Infective endocarditis following mitral valve replacement can occur and further lead to the development of a pseudoaneurysm. We describe a case of left ventricular aneurysm in the setting of infective endocarditis following mitral valve replacement and present radiologic images from various modalities detailing the major findings.

## Introduction

The incidence of bacterial endocarditis has been reported as 2.6 to 7 cases per 100,000 per year in developed countries with a median age of 58 years [1]. Bacterial endocarditis is a concerning find with it being estimated that more than 50% of patients with mitral endocarditis will require mitral valve surgery [2]. One of the most feared complications of mitral valve replacement is left ventricular pseudoaneurysm (LVP). LVP is a rare complication that can result from cardiac surgery as well as myocardial infarction, trauma, and infection [3]. LVP is defined as a rupture of the cardiac left ventricle myocardium contained by adherent pericardium or scar tissue [3-5]. LVP is one of the most feared complications of mitral valve replacement and has a reported mortality rate of 57% to 86% [6,7]. The overall incidence of LVP following mitral valve replacement has been estimated to be 0.8% [8]. One of the greatest risk factors for LVP is age greater than 60 as the cardiac tissue becomes weaker [6]. Other risk factors include female sex, infective endocarditis, and mitral annular calcification [6,8]. One dangerous location for LVP is the atrioventricular groove. Atrioventricular groove disruption (AVGD) is a rare and lethal complication of mitral valve replacement with a reported incidence rate between 0.42% and 1.2% [8,9]. We present a rare case of both LVP and AVGD in a patient complicated by mitral valve endocarditis and subsequent mitral valve replacement.

## Case presentation

**Figure 1:**
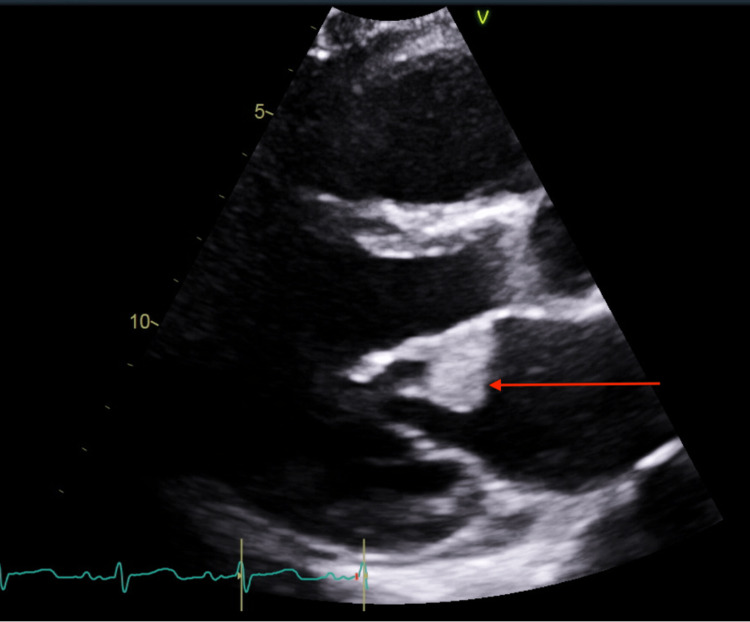
Transthoracic echocardiogram parasternal long-axis view. The red arrow indicates 2.0 × 2.6 cm fixed mitral valve vegetation with associated sclerosis.

**Figure 2:**
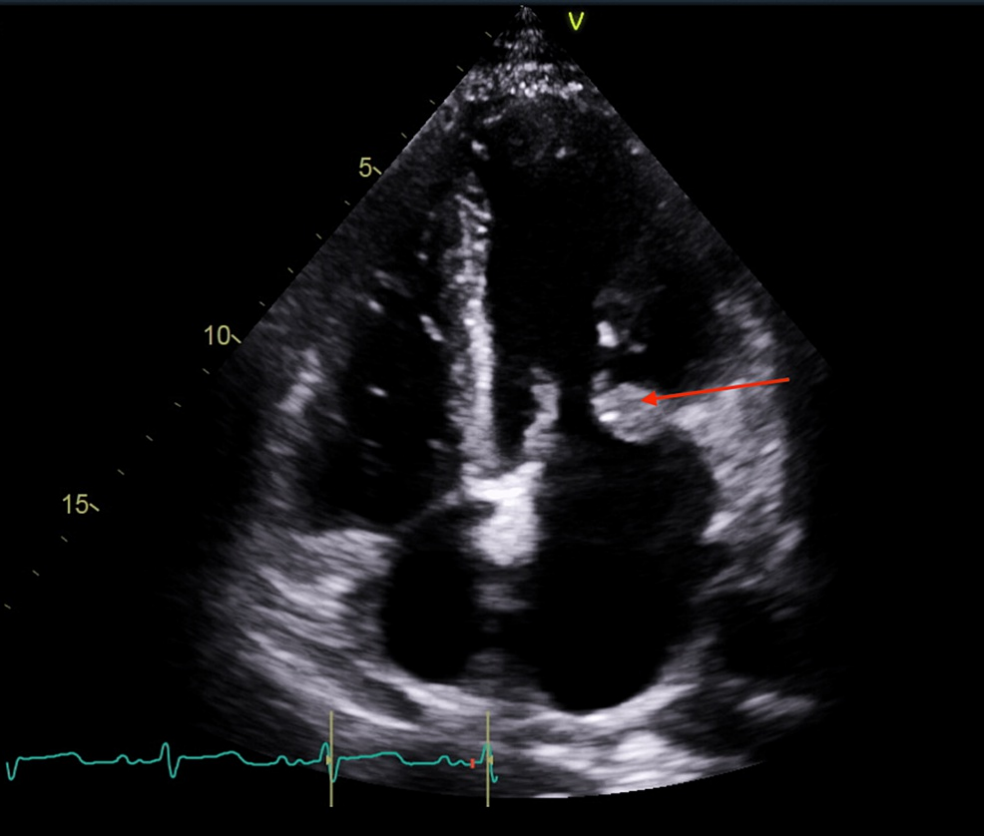
Transthoracic echocardiogram apical four-chamber view. The red arrow indicates 2.0 × 2.6 cm fixed mitral valve vegetation in apical four-chamber view.

A 50-year-old female patient with a past medical history of systemic lupus erythematosus (SLE), interstitial lung disease, myositis, and Raynaud’s syndrome presented to the emergency department of a university hospital with complaints of shortness of breath, chills, fever, and diarrhea. Blood cultures were positive for group B streptococcus and the patient was subsequently treated with ceftriaxone for 35 days. Transthoracic echocardiogram at that time revealed 2 × 2 × 2.6 cm fixed vegetation of the mitral valve with mitral valve sclerosis and significant mitral regurgitation (Figures 1, 2). Subsequent transesophageal echocardiography demonstrated complex mitral annulus calcification, valve sclerosis, severe mitral regurgitation, severe left atrium dilation, and multiple large vegetative masses attached to the upstream lateral portion A1 scallop of the anterior mitral valve annulus and leaflet consistent with complex annular abscess.

The patient underwent subsequent mitral valve replacement, mitral valve annulus repair, and left atrial appendage exclusion with a 40 mm Atriclip. During the procedure, extensive friable vegetation involving the anterior leaflet was noted with prolapse and a leaflet fenestration. The posterior annulus was necrotic and reinforced with Magna Ease Mitral 29 mm bioprosthetic valve. The anterior and posterior leaflets and vegetations were resected.

**Figure 3:**
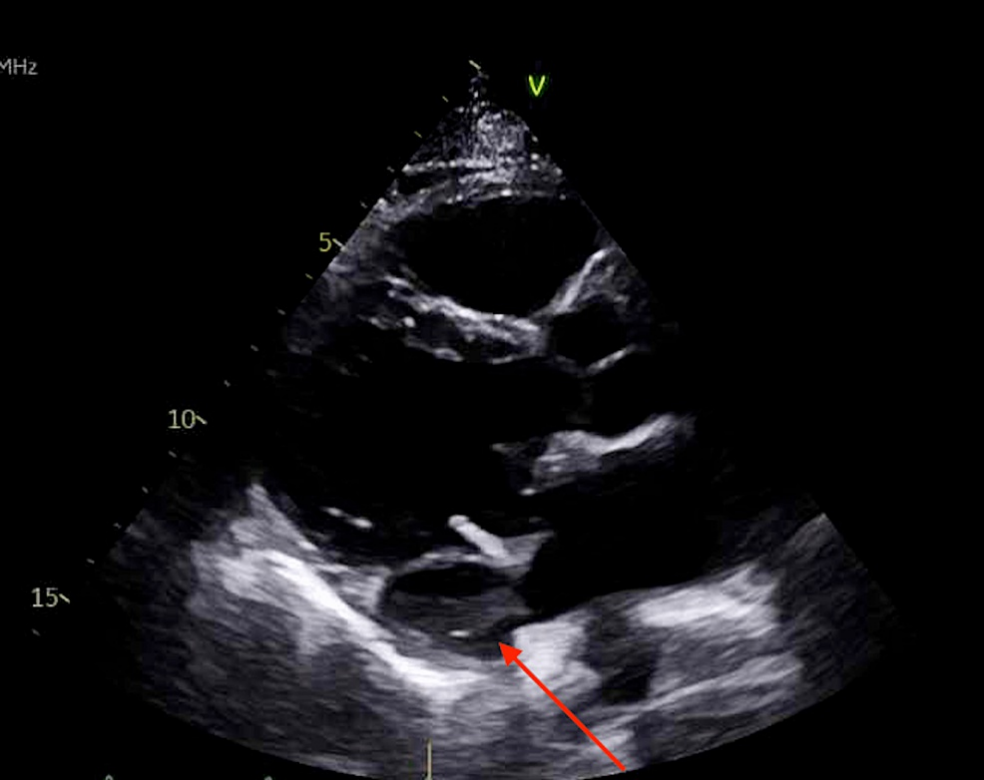
Transthoracic echocardiogram three months after mitral valve replacement. The red arrow indicates left ventricular pseudoaneurysm formation noted on transthoracic echocardiogram three months after mitral valve replacement.

**Figure 4:**
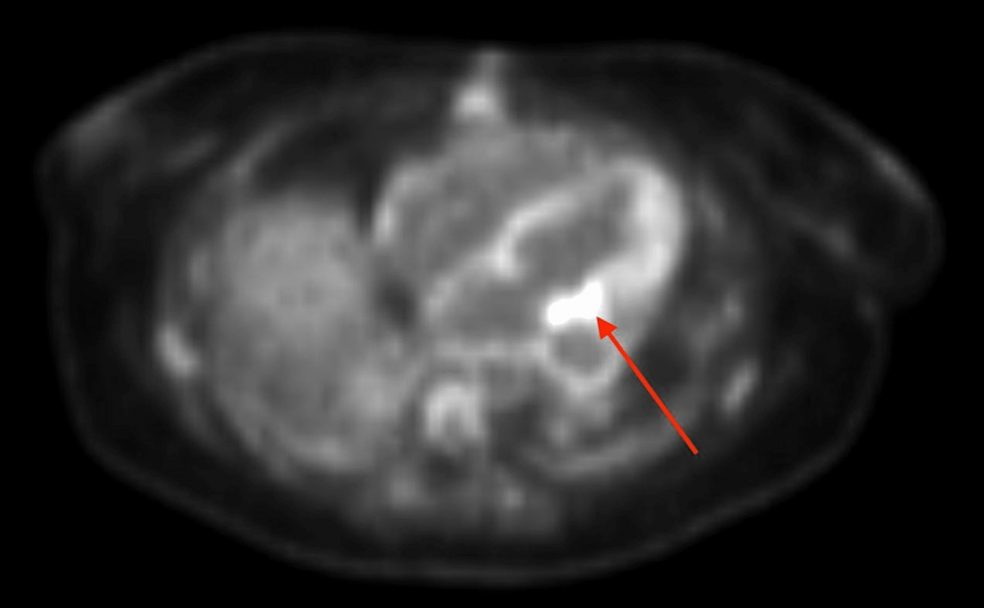
Positron emission tomography scan. The positron emission tomography scan conducted after echocardiography identified a mass along the superior aspect of the mitral annulus and left atrial appendage clip. The red arrow indicates increased uptake of fluorodeoxyglucose, which indicated an infectious source for mitral endocarditis.

**Figure 5:**
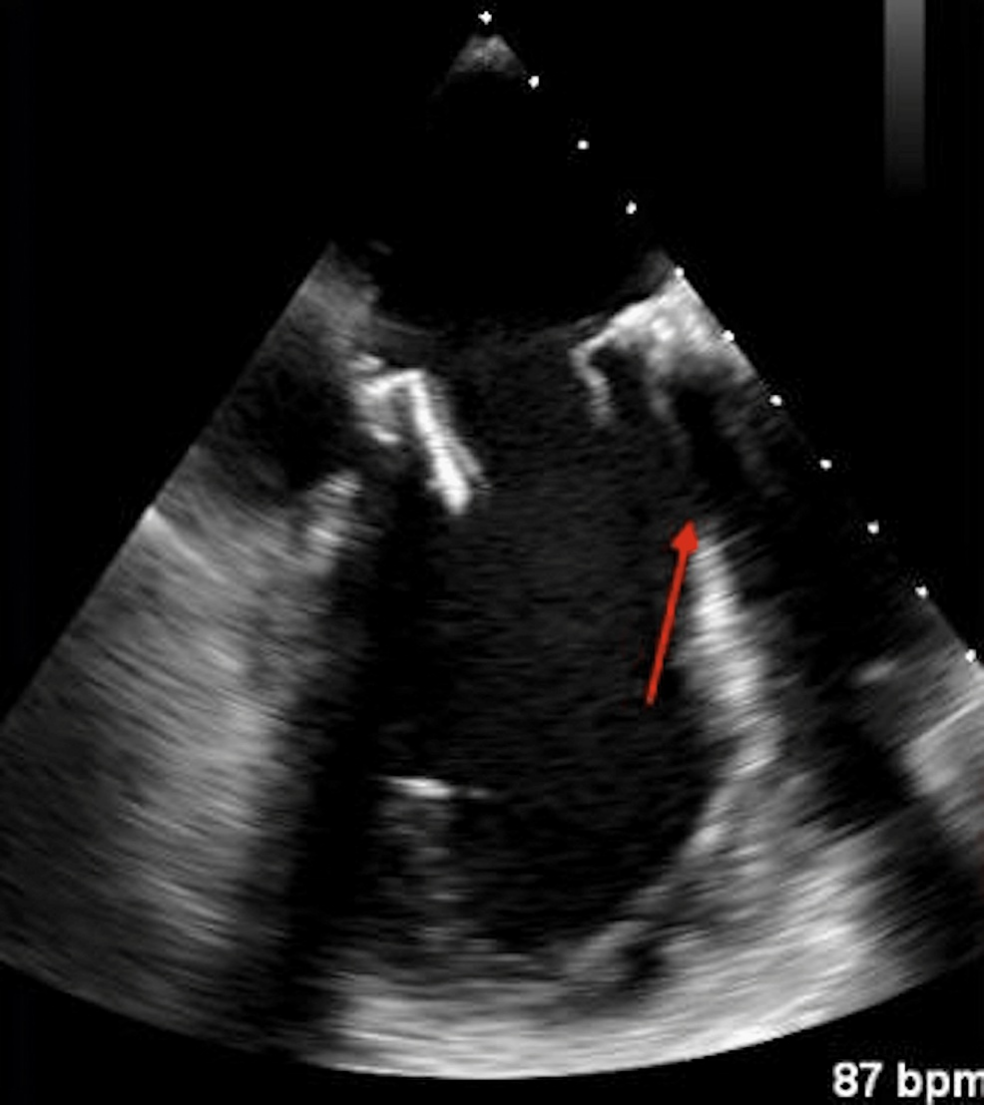
Transesophageal echocardiogram. The red arrow demonstrates a submitral anteroposterior to sublateral left ventricular pseudoaneurysm with a wide neck.

**Figure 6:**
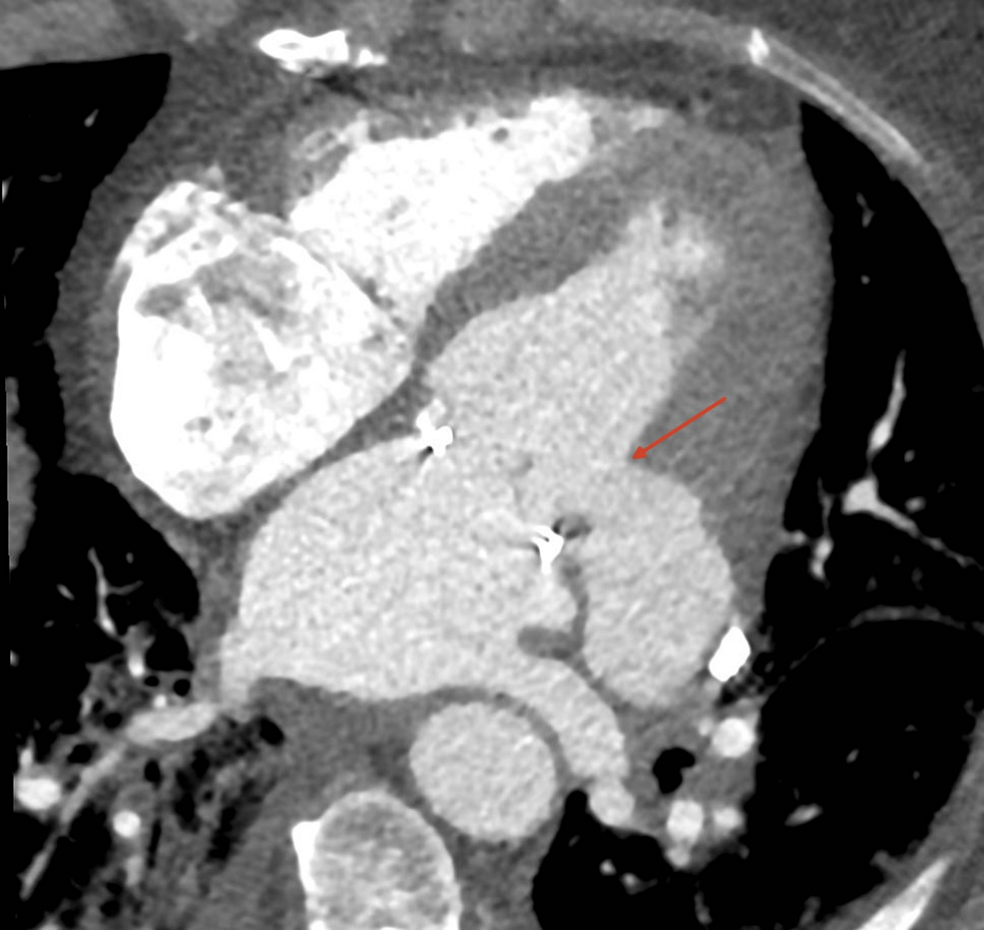
Cardiac computed tomography in a four-chamber view. The red arrow indicates the neck of the left ventricular pseudoaneurysm distal to the mitral valve prosthesis.

**Figure 7:**
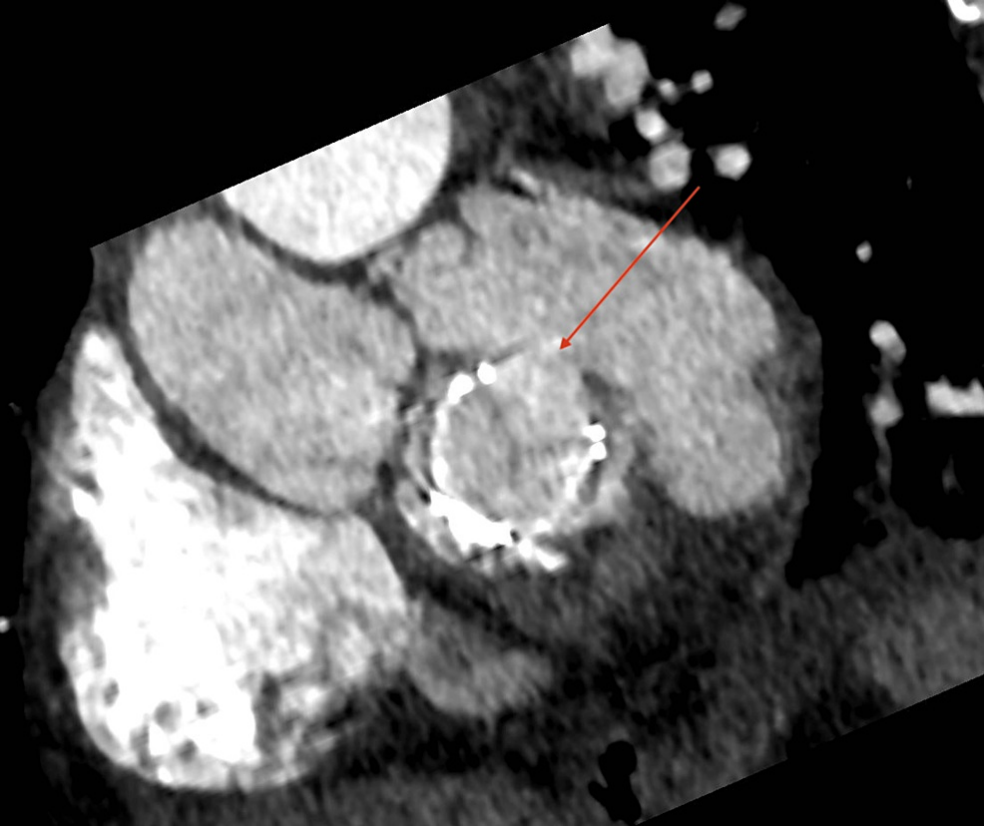
Cardiac computed tomography in the short-axis view. The red arrow shows the opening of the neck of the left ventricular pseudoaneurysm in relation to the mitral valve prosthesis.

**Figure 8:**
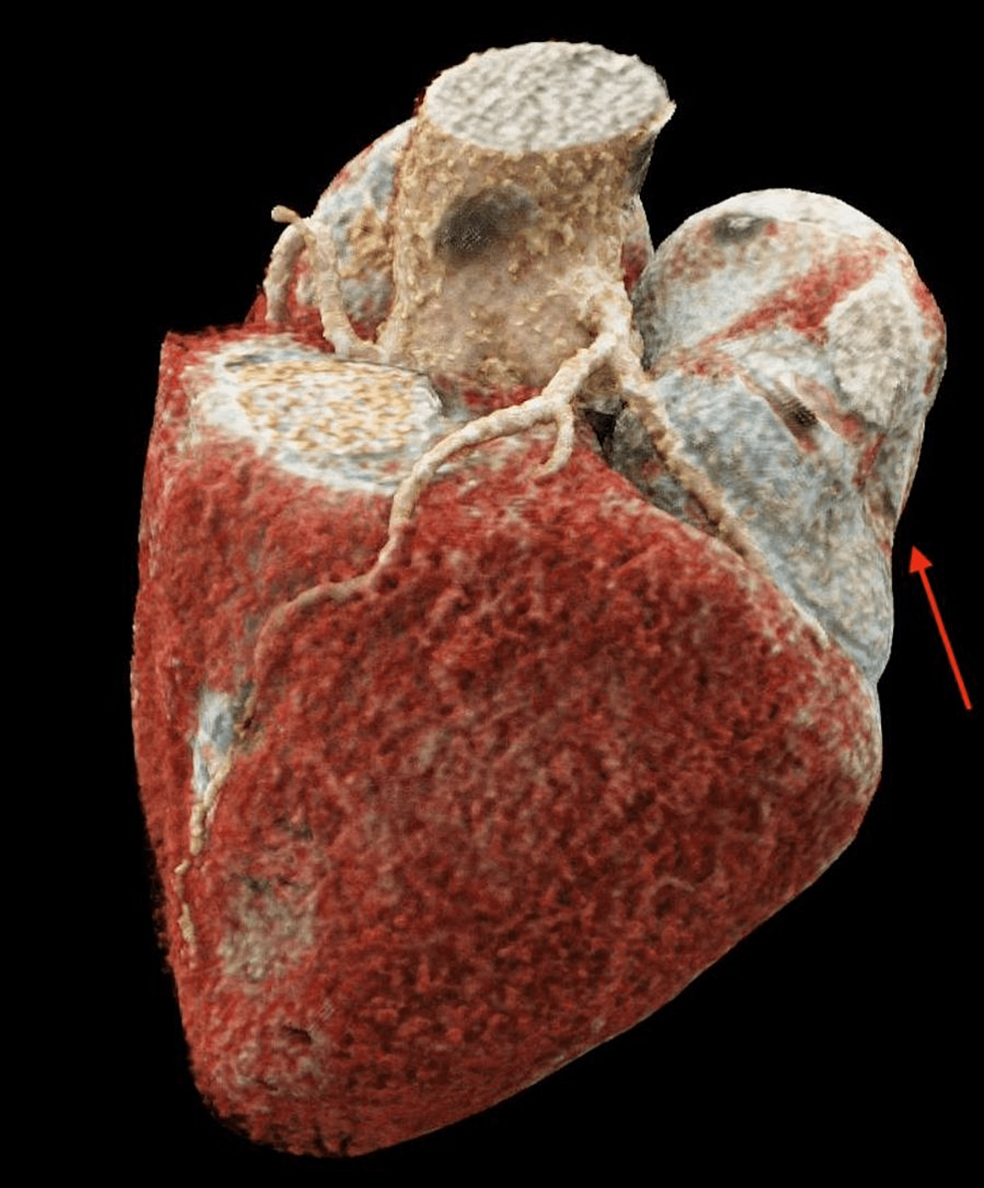
Three-dimensional reconstruction of the patient’s heart and pseudoaneurysm during diastole. The red arrow illustrates the concave nature of the left ventricular pseudoaneurysm during diastole.

**Figure 9:**
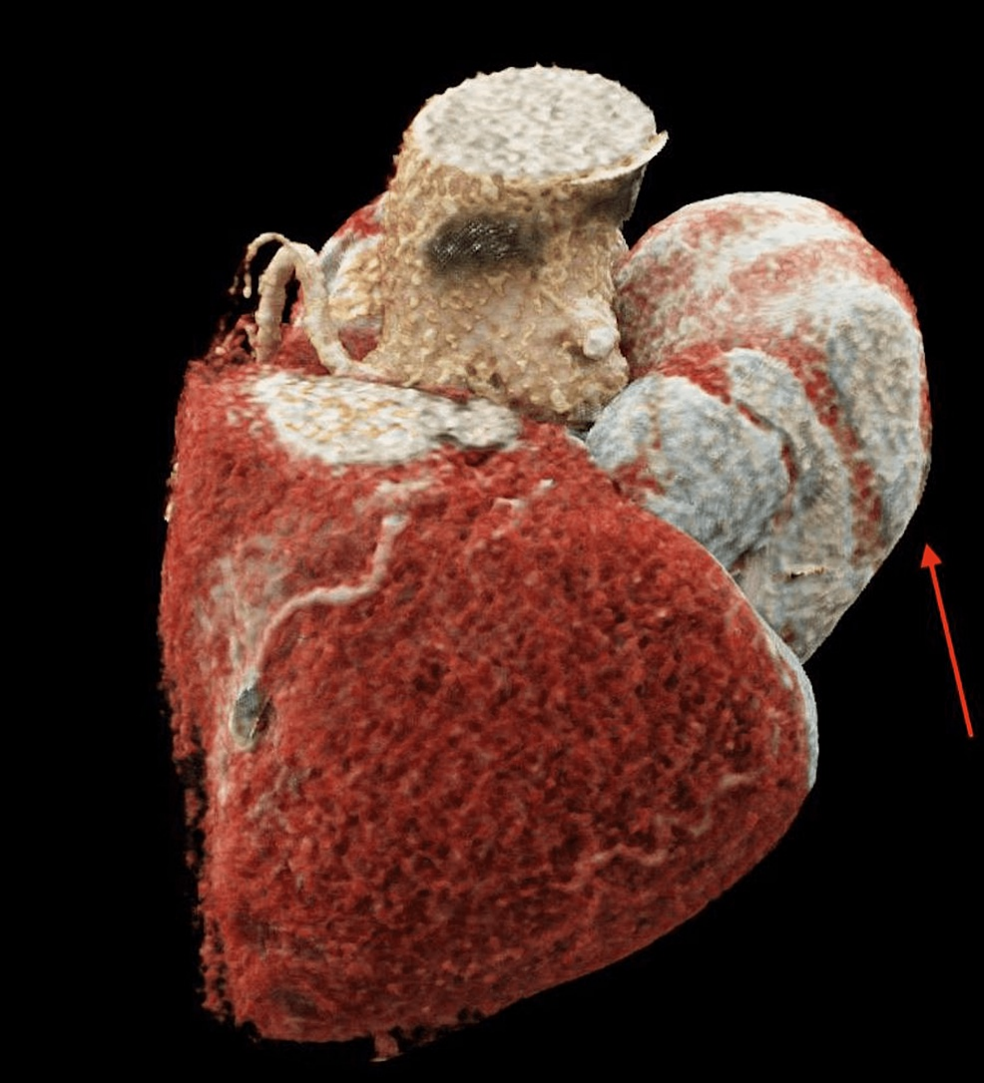
Three-dimensional reconstruction of the patient’s heart and pseudoaneurysm during systole. The red arrow illustrates the convex nature of the left ventricular pseudoaneurysm during systole.

Approximately three months after mitral valve replacement, routine transthoracic echocardiography identified a potential left ventricular pseudoaneurysm (Figure 3). A myocardial positron emission tomography (PET) scan then demonstrated focal marked fluorodeoxyglucose uptake along the superior aspect of the mitral annulus and left atrial appendage clip, concerning for an infectious/inflammatory process (Figure 4). Subsequent transesophageal echocardiogram noted a large submitral anterolateral to posterior pseudoaneurysm (Figure 5). The patient was hospitalized for further evaluation. Blood cultures were collected, and the patient was started on empiric vancomycin and cefepime. Troponin and brain natriuretic peptides were unremarkable. The patient was then admitted to the Cardiology service for further management. The patient underwent computed tomography (CT) cardiac angiogram, which demonstrated a large pseudoaneurysm, measuring up to 6.9 × 3.2 cm, with a wide neck measuring up to 3.6 × 1.4 cm. The LVP arose from the submitral region involving both the superior and lateral aspects of the ventricular wall (Figures 6, 7). The pseudoaneurysm bordered on the left anterior descending artery origin and encased the circumflex artery, which was patent without significant stenosis. The pseudoaneurysm also abutted the left coronary cusp and the left inferior pulmonary vein. There was an interval change in the size of the pseudoaneurysm during diastole and systole, indicating an open communication with the left ventricle and the pseudoaneurysm (Figures 8, 9). Given the history of infective endocarditis of the mitral valve and increased radiotracer uptake on the PET scan, there was a concern for mycotic pseudoaneurysm and AVGD. The patient was transferred to a nearby outside hospital. Mechanical mitral valve replacement and left ventricular basal pseudoaneurysm repair with a bovine pericardial patch were performed. The patient was taken to the intensive care unit postoperatively in stable condition. When the patient was hemodynamically stable, she was transferred to the step-down unit and discharged.

## Discussion

AVGD is a rare complication of mitral valve replacement and may present as an LVP. This potentially lethal complication is a medical emergency where early imaging can lead to a timely diagnosis and proper treatment. AVGD has an estimated mortality rate of 69%-100% [6,9,10]. Historically, posterior ventricular rupture can be divided into three types [8,11,12]. Type I is a rupture at the posterior atrioventricular sulcus or groove. Type II is a rupture of the posterior left ventricle located at the base of the papillary muscle. Type III is a rupture located somewhere between the anatomical locations of type I and II. The patient in this case report presented with a type I rupture most likely due to calcification and infection of the mitral valve.

There are several risk factors associated with developing a cardiac pseudoaneurysm. These risk factors include female gender, older age, history of hypertension, and cardiac tissue damage. The second leading cause of LVPs is cardiac surgery [13]. In particular, mitral valve replacement surgery typically has the highest rate of LVP formation of 0.8% of all cases [8]. While the etiology of cardiac pseudoaneurysms can be multifactorial, including post-infarction, post-surgical, or traumatic causes, their association with autoimmune diseases, particularly SLE, has been rarely reported. The pathogenesis of cardiac pseudoaneurysm formation in SLE is complex and likely involves several interrelated factors. Endocardial and myocardial inflammation, microvascular abnormalities, and immune complex deposition are proposed mechanisms [14]. Inflammatory processes may weaken the ventricular wall, predisposing it to rupture. Additionally, the presence of accelerated atherosclerosis, vasculitis, and immune-mediated damage may contribute to the development of pseudoaneurysms. SLE is known to cause damage to multiple organs in the body including the heart. SLE can affect the pericardium, myocardium, coronary arteries, valves, and the conduction system [14]. Approximately 15% and 25% of patients with SLE will experience endocarditis and pericarditis, respectively [14]. This elevated risk of endocarditis and other cardiac abnormalities requires additional attention. For patients with increased risk factors for LVP, more aggressive follow-up imaging may be necessary to identify potential complications.

Prompt diagnosis is critical to providing potentially life-saving treatment to patients presenting with AVGD. The most commonly used techniques to diagnose a cardiac pseudoaneurysm include transthoracic echocardiogram, transesophageal echocardiogram, CT, angiography, and chest X-ray. First-line diagnostic imaging for cardiac pseudoaneurysms is transthoracic echocardiogram because it is non-invasive and carries a significantly lower cost compared to CT and angiography. In 85%-90% of patients with an LVP, an abnormality is noted on Doppler echocardiography [5]. However, a definitive diagnosis can only be made 28%-31% of the time [5]. Therefore, suspected anomalies detected on ultrasound require further follow-up imaging and workup. The transthoracic echocardiogram images presented in this case report were able to clearly show an abnormal cardiac process that was likely a pseudoaneurysm. Further imaging was able to confirm these findings and allow the patient to receive proper treatment. Utilizing cardiac CT, we constructed a three-dimensional model of the pseudoaneurysm. This model demonstrated both the notable size of the defect as well as the open connection with the left ventricle during systole and diastole.

One of the more unique techniques to identify potential infections is PET. In this case report, a myocardial PET scan helped identify an endocardial mass from a likely infectious process due to the increased metabolic activity of the mass. The American College of Cardiology and the American Heart Institute 2020 guidelines now include PET scans as part of the workup for patients with suspected infective endocarditis [15].

AVGD is a potentially fatal complication of mitral valve replacement that must promptly be addressed. The only definitive treatment for a cardiac pseudoaneurysm is surgery to repair the defect and maintain the structural integrity of the heart. The surgery repair itself carries a significant risk of mortality (20%-36%) [3]. However, the risk of not treating the defect carries a greater risk to the patient. One option to treat patients who are not good surgical candidates is percutaneous embolization. This treatment involves inserting a catheter endovascularly into the left atrium and ventricle where embolization coils can be utilized to close off the pseudoaneurysm. However, this procedure is contraindicated in patients with active endocarditis and/or the presence of left atrial thrombus [3]. Based on the size of the pseudoaneurysm and the infection of the mitral valve replacement, surgical management was necessary.

Traditionally, repair of AVGD has been via an “internal” approach. This involves the removal of the original prosthesis, placing a patch from within the cardiac cavity to reconstruct the atrioventricular groove, and then reimplanting a new prosthesis. This technique has reported mortality rates of 69%-100% [6,9,10]. More recently, there has been the development of an alternative “external” approach for the rescue of type 1 AVGD with reported mortality rates of 15% [8]. The external approach involves analysis of the full patient scenario. The internal approach may be better suited for younger patients in overall better health, while the external repair provides a valid option for patients who may not tolerate an invasive surgical intervention [8]. Cardiac pseudoaneurysms have a 30%-45% chance of rupture with a subsequent high risk of mortality [16]. The patient in this case report underwent a full mitral valve prosthesis replacement and intracardial bovine patch.

## Conclusions

This case report included multiple images from various imaging modalities showing a large LVP resulting from AVGD in the setting of mitral endocarditis. Mitral endocarditis itself is a major risk factor for mitral valve surgery. Multiple risk factors are associated with developing an AVGD and subsequent LVP after mitral valve replacement surgery. In this case report, SLE is suggested as an additional possible risk factor for cardiac pseudoaneurysm formation that may require more detailed follow-up. Continued follow-up of all patients following mitral valve replacement can identify complications such as LVP. A transthoracic echocardiogram is a quick, accurate, and non-invasive exam to evaluate patients. PET scans along with traditional imaging techniques of ultrasound and CT scans can help to identify the aneurysm itself as well as potential complications such as infection.
